# The Impact of COVID-19 Pandemic on Medical Doctors’ Work-Family Balance at German University Clinics

**DOI:** 10.3390/healthcare10020227

**Published:** 2022-01-25

**Authors:** Caroline Beutner, Anja Lipschik, Luise Erpenbeck, Jason Holsapple, Michael P. Schön, Hedwig Stanisz

**Affiliations:** 1 Department of Dermatology, Venereology and Allergology, University Medical Center Göttingen, 37075 Göttingen, Germany; caroline.beutner@med.uni-goettingen.de (C.B.); luise.erpenbeck@ukmuenster.de (L.E.); Jason.holsapple@ukmuenster.de (J.H.); michael.schoen@med.uni-goettingen.de (M.P.S.); 2 Equal Opportunities Representatives, University Medical Center Göttingen, 37075 Göttingen, Germany; anja.lipschik@med.uni-goettingen.de; 3 Department of Dermatology, University Clinic Münster, 48149 Münster, Germany; 4 Lower Saxony Institute of Occupational Dermatology, University Medical Center Göttingen, 37075 Göttingen, Germany

**Keywords:** COVID-19, university medicine, female doctors, work-family balance, childcare

## Abstract

The measures taken to cope with the COVID-19 pandemic by governments worldwide have vast consequences on all areas of life. To assess the impact of the COVID-19 pandemic on long-term career development, we evaluated the work-family balance of medical doctors at nine German university clinics. The results indicate a severely disturbed work-family balance, which was mostly due to insufficient childcare, based on restrictions in school operations and childcare. Despite the newly created emergency childcare options, aiming to ensure the functioning of the “systematically important” professional groups, medical doctors feel that they are not sufficiently supported by the measures taken by local governments. Women, in particular, see their professional development at risk. Our results underline that proper and flexible childcare is essential for the career advancement of female medical doctors and is particularly important in times of crises such as the current COVID-19 pandemic. At university medicine clinics, increased work time flexibility and optimized schooling and childcare are needed to promote the career development of female as well as male medical doctors in the early stage of their careers.

## 1. Introduction

The COVID-19 pandemic has resulted in severe restrictions in many areas of life. Initially, the focus was on the immediate health threat and the containment of the pandemic. In Germany, the central efforts of the state and society were meant to counteract an overload of the health system caused by the pandemic [1]. Closures of schools and childcare facilities, as well as restrictions on social and economic life, were the result. Extensive restructuring in the healthcare sector was also carried out in order to be able to maintain capacities for patients with COVID-19 [2]. These and other restrictions had an effect in terms of containing the pandemic. Contemporaneously, however, there are increasing long-term consequences for our society [3]. In order to maintain the ability to work for “systemically important” occupational groups during the lockdown, emergency care was set up for children and adolescents [2,3].

Women and children are considered to be the group that has been most severely socially affected by the consequences of the pandemic [4]. More than 70% of children and adolescents feel stressed by the COVID-19 crisis [5]. Compared to men, women seem to have a slightly lower vulnerability and mortality to severe acute respiratory syndrome coronavirus 2 (SARS-CoV-2) [6]. In addition to the medical aspects [7,8,9], the current pandemic has other serious consequences [3,10]. These consequences reveal striking differences between the sexes with medium-to-long-term negative influences on the implementation of gender equality [11]. The restrictions on external childcare existing for months, as well as the unpredictability of future measures in this area, are likely to have a much stronger impact on women who do most of the “family work”. This leads to a forced reduction in working hours and a restriction of job market opportunities [6,12].

The gender care gap, which is the different time expenditure for unpaid care work for men and women, is aggravated by the pandemic. The gap in terms of equality between men and women is widening [12]. The compatibility of work and family, i.e., work-family balance—in which effectiveness and satisfaction is experienced in both salient fields through equal involvement—is seen as questionable [13]. Meanwhile, work-family stress—i.e., the conflict of the work role and the family role, compromising the performance and satisfaction in one or the other area—negatively influences the feeling of work-family balance [14,15]. Surveys on these topics have high response rates, but are also increasingly answered by women [16]. According to the Federal Institute for Population Research, family work also increased somewhat for men during the lockdown [17]. However, terms such as “backlash” or “retraditionalization” are used to describe the role of working mothers during the COVID-19 pandemic [16,18].

The gender gap is a scientifically proven fact in the medical sector, regardless of the COVID-19 pandemic. Although there are more female than male medical students, the number of women in leading academic medical positions is still by far comparably low. This fact is accompanied by a gender pay gap, further illustrating the gender inequalities in academic medicine [19]. There are increasing reports that the professional development of female doctors and scientists, in particular, is being significantly hindered by the pandemic [20]. This applies especially at university medical clinics, in which career development is based on success in day-to-day healthcare, as well as in research. Efforts concerning parity and the achievements of recent years are being undone by the crisis [11,18]. There are calls from various areas of interest to analyze the effects of the pandemic on a gender-specific basis [21].

University hospitals are considered one of the most important and indispensable pillars of healthcare in the current situation. For the first time in Germany, in this study we intended to depict the status of the work-family balance among doctors in German university hospitals in the context of the COVID-19 pandemic. This was achieved using a newly developed structured questionnaire, designed to characterize the areas of tension in a profession that has been structurally relevant since the first hour of the pandemic, in which attendance is predominantly mandatory and at the same time in which family obligations cannot be neglected. By focusing on the assessment of the perceived stress and the perception of the political measures implemented to secure a systemically important workforce, essential aspects of the work-family conflict have been brought into the light by the COVID-19 pandemic. Their analysis from the perspective of gender equality enables the deduction of suggestions for improving the work-family balance, as well as measures to promote gender equality during the COVID-19 pandemic and beyond.

## 2. Materials and Methods

### 2.1. Data Collection

A structured 18-point questionnaire was designed to depict the areas of the professional and family life of medical doctors. It was organized in such a way as to collect data about gender, the number of children and qualifications, as well as the initial professional and family situation. In addition, there was a differentiation between workload and the combination of family obligations and workload. Furthermore options and suggestions for optimization were recorded and the effects of the current overall situation on each respondent’s own professional career were assessed. The questions were all closed questions with given possibilities of answer, with some questions having several possibilities for responses. Finally, the participants were able to voluntarily name their specialist area and add other aspects of importance in free text. However, this last part did not undergo analysis but served as a source of additional information to avoid missing central aspects not addressed in the questionnaire.

### 2.2. Data Analysis

The survey and the evaluation were carried out via the Evasys survey portal, anonymously and in accordance with data protection regulations. The results were analyzed automatically through the survey portal as all questions were closed. A statistical analysis of central aspects was carried out, using one-sided exact tests according to the Fisher test function in the statistical programming environment R (version 3.6.0, R Foundation for Statistical Computing, Vienna, Austria).

### 2.3. Sample

The survey was explicitly aimed at parents with children requiring care in the medical service at university hospitals. The invitation was sent via e-mail to all medical doctors at the University Medical Center Göttingen (UMG) and to eight other university hospitals nationwide: Bochum, Dresden, Duesseldorf, Freiburg, Greifswald, Kiel, Lübeck and Mainz. Contact was made via Equal Opportunities Representatives, who are connected via the Federal Conference of Equal Opportunities Representatives at Universities (Bundeskonferenz der Frauen- und Gleichstellungsbeauftragten an Hochschulen e.V (Bukof)), as well as through the German Medical Women’s Association. In the following, we will refer to the participating university clinics nationwide as Bukof clinics. The survey started on 14 May 2020, a reminder was sent by email on 2 June 2020 and the survey was closed on 5 June 2020.

## 3. Results

A total of 656 medical doctors from the nine university clinics (UMG and eight Bukof clinics) took part in the survey. Due to the high participation rate and the balanced gender ratio, the UMG collective was analyzed separately and compared with the nationwide collective of the Bukof clinics.

### 3.1. Initial Professional and Family Situation

At the UMG, the survey was sent to the entire “doctors distribution list”, comprising 1270 doctors. The survey was explicitly aimed at parents with children requiring care. At the UMG there were 447 medical employees with children under the age of 18 (48.7% women). One hundred eighty-nine colleagues took part, which corresponds to a participation (response rate) of 42.2%. The balanced participation of both genders (48.7% women and 51.3% men) was particularly noticeable, whereas women (82.5%) clearly comprised most of the 467 participants nationwide (Table 1). Around a third of the participants worked each as interns, specialists or senior physicians. A large number of male colleagues worked full-time, whereas the proportion of female doctors working full-time was significantly lower (Table 1).

The focus of activities of the doctors at the UMG and the Bukof clinics was predominantly conservative. Both collectives included employees from the “COVID-19 team” (colleagues who were primarily involved in the treatment of patients infected with SARS-CoV-2). Seventy percent of the doctors participating in the UMG and 72% of the doctors nationwide stated that they had at least two children (Table 1).

### 3.2. Initial Situation and Change in Childcare during the COVID-19 Pandemic

Most of the doctors (UMG: 82.6% of the male doctors and 69.9% of the female doctors; Bukof clinics: 78.9% of the male doctors and 66.3% of the female doctors) did not make use of private childcare before the start of the COVID-19 pandemic. They were therefore largely dependent on public childcare facilities to be able to carry out their professional activities. After the start of the pandemic, only a small proportion of the doctors were able to top up their existing private childcare or to initiate new ones in order to cover parts of the care or off-peak times (UMG: 15.1% of the male doctors, 19.2% of the female doctors; Bukof clinics: 14.5% of the male doctors, 21% of the female doctors). Emergency childcare (including public care facilities and company emergency childcare) was offered to 75.4% (UMG) and 72.2% (Bukof clinics) of the doctors; however, this only covered 22.6% (UMG) and 28.6% (Bukof clinics) of the working hours (proportions about the same for both sexes). We found that 66.7% of the doctors at the UMG and 69% nationwide felt that they were not adequately supported by the childcare modalities created.

### 3.3. Work and Family Stress during the COVID-19 Pandemic

**Figure 1:**
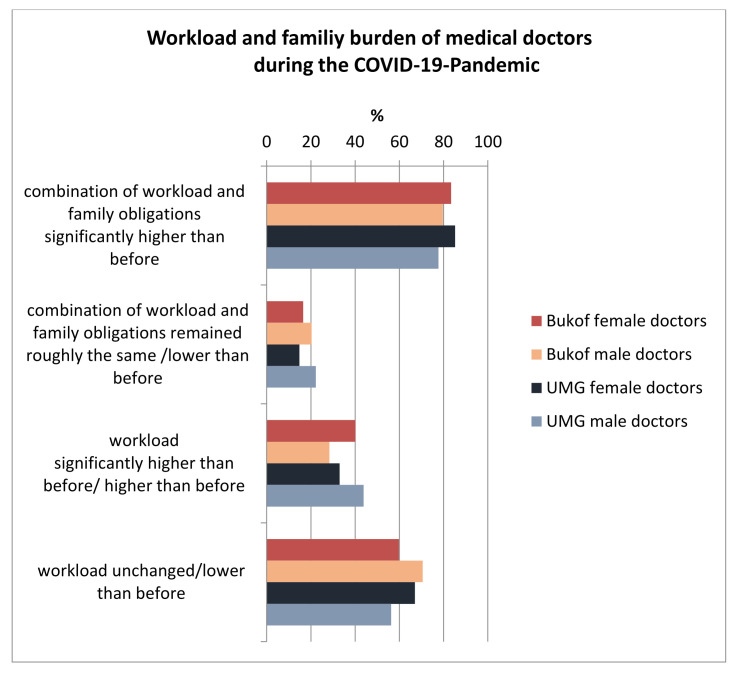
Assessment of the combination of private and professional stress, as well as the purely professional stress, stratified by the gender of the doctors (%) of the University Medical Center Göttingen (UMG) and university clinics nationwide (Bukof clinics).

The majority of the doctors in both groups (>78%) rated the combination of work and family stress during the COVID-19 pandemic as higher or significantly higher than before. This again primarily applied to the women in both groups, with 85.2% (UMG) and 83.4% (Bukof clinics) (Figure 1). This result is particularly remarkable because the pure occupational exposure of the doctors was assessed as unchanged or lower by 56.3% (UMG) and 71.6% (Bukof clinics) (Figure 1).

### 3.4. Adjustment of Doctors’ Working Hours to the Changed Childcare Situation during the COVID-19 Pandemic

**Figure 2:**
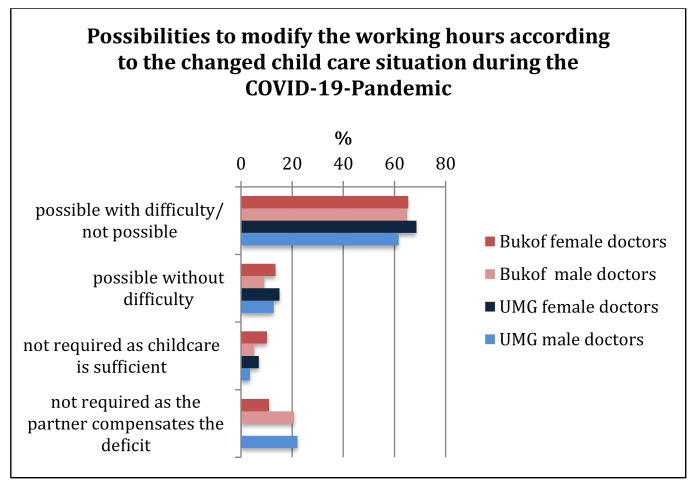
Possibility or necessity of adjusting working hours due to the changed childcare situation for doctors in the COVID-19 pandemic.

The proportion of doctors in both groups with difficult or no options for working-time adjustments was similarly high (>60%). Only a small proportion (<10%) stated that working-time adjustments were not necessary due to their having sufficient childcare. Compensation by the partner was more likely for male doctors (UMG 22.1%, Bukof clinics 20.8%) than for female doctors (UMG: 9.3% Bukof clinics 11%) (Figure 2). Furthermore, 15.4% of the part-time workers at UMG and 17% of the part-time workers nationwide would have volunteered to increase their working hours, if this had been necessary due to the pandemic situation in the clinics.

### 3.5. Options to Optimize the Support of Medical Doctors in the Pandemic Situation

**Figure 3:**
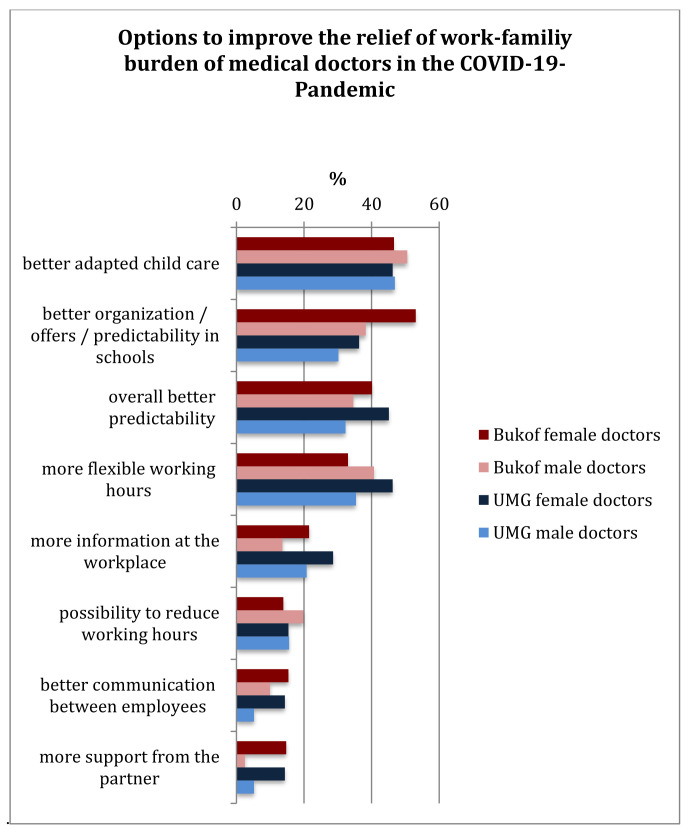
Options to improve the compatibility and relief of the work-family tension of doctors in the COVID-19 pandemic (multiple answers possible).

Organizational aspects were also in the foreground for both collectives, such as improving childcare and support from schools, as well as flexibility in terms of working hours and long-term planning. The focus was not on the possibility of reducing working hours; more freedom and flexibility was desired regarding working hours. Support through a stronger commitment from partners tended to be in the background, but here mostly female doctors from both collectives wished for better support (Figure 3).

### 3.6. Impact on Career

**Figure 4:**
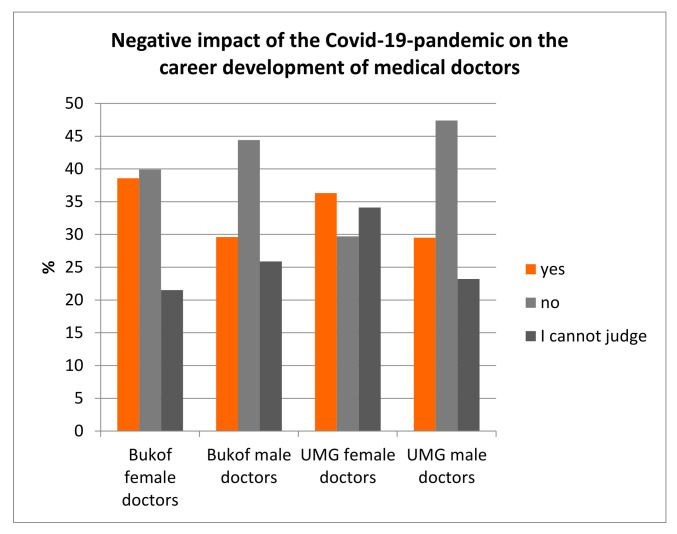
Doctors’ assessments of the extent to which the COVID-19 pandemic was having a negative impact on their own professional career development. This was statistically significant in a comparison of female and male doctors from the UMG (*p* < 0.041).

Thirty-three percent of those surveyed at the UMG stated that their careers were suffering under the current conditions; nationwide this was 37%. If one stratifies the data according to the gender of the UMG participants, it is noticeable that 36.3% of the female doctors stated that their careers were suffering from the changed conditions, compared with only 29.5% of the male doctors (Figure 4). Looking at the individual subgroups, 42.1% of the female interns felt that their career development was impaired; among the male interns, it was 31.8%. Moreover, 27.3% of the female specialists and 23.8% of the male specialists saw a negative impact on their professional development. For senior physicians it was 40% for the females, compared with 31.4% for the males. Nationwide, a comparable picture emerged: 38.6% of female doctors stated that their careers were suffering in the current situation, whereas it was 29.6% for male doctors (Figure 4). In the subgroups, it was 41.2% for female interns versus 36.8% for male interns, for specialists it was 30.1% females and 29.6% males and for senior physicians it was 48.3% females versus 21.2% males. Overall, the proportion of respondents who did not fear a loss of their career was high among the male doctors in both groups (UMG: 47.4% and Bukof clinics: 44.4%) compared to the female doctors (UMG: 29.7% and Bukof clinics 39.9%). Female doctors saw themselves as impaired in their career development, especially in the early career phases of the internship, but also in the later career-deciding phases.

## 4. Discussion

Regardless of their profession, a high level of stress has been reported for many parents during the pandemic [16,17]. This is particularly true in healthcare [22]. The assumption that around one third of all medical professionals at German university hospitals are parents of children requiring care goes along with our numbers. This significant proportion of employees was exposed to broad and long-lasting stress during the COVID-19 pandemic. However, this was not basically due to the workload, which for most of the colleagues was not fundamentally different from normal operations. A recent survey by the Marburger Bund yielded similar results: 82.4% of the 8707 members saw their workload in March 2020 as decreased or unchanged [23].

Overall, it is becoming clear that the political requirements for creating emergency care and its practical implementation are insufficient. Their effectiveness should be reconsidered promptly and corrective measures should be taken. This is of particular importance in order to not further undermine the already insufficient measures to promote equality and a work-family balance for female doctors.

For most doctors, emergency care does not adequately reflect working hours, even if they work part-time. Even the somewhat extensive, newly-created emergency childcare offered within the companies, which were often highlighted very positively in the free text comments in this study, cannot provide a sufficient remedy here, as many children are not cared for in the companies. Only a small number of doctors were able to achieve flexibility through private childcare, the financing of which was not recorded. The majority of the doctors were completely dependent on emergency childcare from public institutions. It is not surprising that the majority (>78%) estimated the professional and private double burden to be higher or significantly higher than before the COVID-19 pandemic. This problem is exacerbated by the combination of inadequate childcare and little or no possibility of adapting working hours in an occupation requiring attendance. In both groups (Göttingen University Medical Center and nationwide university clinics), similar demands for improvement were made regardless of gender. The support factors favored in this survey were better-adapted childcare and schooling, as well as flexible options for organizing work and taking advantage of offers of assistance. These are already part of the recommendations for reducing stress and psychological distress for health professionals during the current COVID-19 pandemic [22].

Male doctors also rated the childcare situation as inadequate and the double burden as high. However, female doctors were more affected, due to their higher burden of family care-work [12,18]. Due to different levels of earned income, women are more likely to reduce their working hours. However, this argument does not usually apply to the medical profession, with collectively agreed salaries at university hospitals. This implies that traditional role models may also be decisive [12].

In particular, female interns and senior physicians feel that their career development is more impaired than their male colleagues do. This finding is underlined by the literature: after an analysis of 60,000 articles in international journals, the proportion of scientific articles by female first authors decreased considerably [20]. The hereby-documented reduced “scientific productivity” as a result of the lockdown is likely to have long-term negative consequences for the careers of women, especially in academic medicine [20].

Both female and male doctors desire more flexible working hours so that they can better adjust to the requirements of their families. The focus is explicitly on more flexibility in working hours and not on reducing them. It is well known that the adjustment of working hours is more common and more recognized for female doctors than for male colleagues, who, as our data show, often have even greater difficulties in terms of flexible organization. This becomes much clearer under the intensified conditions of the pandemic, but should also be relevant under normal circumstances.

## 5. Theoretical and Practical Implications

The early phase of the COVID-19 pandemic already revealed the insufficiency of the emergency childcare system created. This is an issue of particular importance as it further undermines the already insufficient measures to promote equality and work-family balance for female doctors. Female doctors are more affected, due to their higher burden of family care-work [12,18], which is still based on traditional role models [12].

The aim of not pushing a health system to its limits includes, in addition to (intensive) medical capacity increases, maintaining the workforce and health of indispensable actors [24,25,26]. This includes providing sufficient support to families through the means of emergency childcare. This is fundamental to ensure the presence and functionality of doctors who are already exposed to psychological stress due to the pandemic situation [27,28,29].

More flexible working hours for both genders and their implementation and acceptance as an explicit option for male colleagues may also be a way of relieving and thus promoting the careers of female doctors.

## 6. Conclusions

Overall, the COVID-19 pandemic demands the adaptability not only of individual employees in the health system but also of employers to maintain the ability of employees to work. The explicit promotion of opportunities for the development of scientific careers for female doctors with families also appears necessary. Adaptable childcare concepts, especially during the COVID-19 crisis, but also beyond it, are still an important component in promoting employees with families.

Gender analyses during the COVID-19 pandemic are useful in order to uncover structural deficits that have become more apparent during the crisis and to make them public. Although our study mainly focuses on the specific aspect of work-family stress during the COVID-10 pandemic, concrete research and knowledge of the deficits offers the opportunity to find new solutions and to transform the initially negative consequences of the pandemic into a structural improvement.

## Figures and Tables

**Table 1:** Characteristics of the surveyed collectives: doctors from the University Medical Center Goettingen (UMG) and university clinics nationwide (Bukof clinics).

Parameter	University Medical Center Göttingen (UMG) Collective I	University Clinics National (Bukof-Clinics) Collective II
Participating medical doctors total (N)	189	467
female (%)	48.7	82.5
male (%)	51.3	17.5
interns (%)	32.0	27.0
specialists (%)	29.3	38.2
senior physicians (%)	38.3	34.4
doctors working fulltime		
female (%)	31.1	48.8
male (%)	91.7	91.4
main working field		
- surgical (%)	26.5	21.4
- conservative (%)	67.7	63.9
- scientific (%)	5.8	14.7
≥2 children (%)	70.0	72.0

## Data Availability

The data presented in this study are available in the article.
